# Outpatient Total Hip and Knee Arthroplasty Performed in a Safety Net Hospital System

**DOI:** 10.5435/JAAOSGlobal-D-21-00117

**Published:** 2021-09-16

**Authors:** Robert Daniel Kay, Adam James Taylor, Erik Yeh Tye, Jason Andrew Bryman, Robert Patrick Runner

**Affiliations:** From the Rancho Los Amigos National Rehabilitation Center, Department of Surgery—Surgical Arthritis, Downey, CA (Dr. Kay, Dr. Taylor, Dr. Tye, Dr. Bryman, and Dr. Runner), and the Department of Orthopaedic Surgery, Medical Center, Harbor-University of California, Los Angeles, CA (Dr. Kay, Dr. Taylor, Dr. Tye, and Dr. Bryman).

## Abstract

**Introduction:**

High-percentage outpatient total joint arthroplasty (TJA) performed in a safety net hospital system has not been described. A rapid recovery protocol (RRP) was instituted at our safety net hospital that allowed eventual transition to outpatient TJA.

**Methods:**

Retrospective review of all primary total knee and hip arthroplasty performed by a single surgeon (RR) using an RRP was performed. The initial cohort of patients was monitored overnight with the goal of next-day discharge (n = 57), and as the RRP evolved, the subsequent cohort of patients had the possibility of same-day discharge (PSDD, n = 61). Outcome measures included the rate of same-day discharge in the PSDD cohort and short-term adverse event rates.

**Results:**

In the PSDD cohort, 86.9% (n = 53) of patients were successfully discharged on the day of surgery, and hospital length of stay was decreased by 17.7 hours (13.5 versus 31.2 hours, *P* < 0.0001). Comparing the next-day discharge and PSDD groups, no significant differences were found in 30-day emergency department visits (5.3% versus 3.3%, *P* = 0.67), 90-day complications (15.8% versus 13.1%, *P* = 0.79), 90-day readmissions (0% versus 3.3%, *P* = 0.50), or 90-day revision surgeries (0% versus 3.3%, *P* = 0.50).

**Conclusions:**

This study demonstrates that the transition to outpatient TJA can be successfully performed in a safety net hospital system without increasing short-term adverse events.

In recent years, there has been a push toward the implementation of outpatient total joint arthroplasty (TJA) programs.^1,2^ Oftentimes, patients who are otherwise appropriate candidates for outpatient TJA receive no medical interventions when admitted to the hospital overnight.^3^ In appropriately selected patients, outpatient TJA has been shown to be safe and effective, resulting in decreased hospital stays and reduced economic burden to the healthcare system without increasing complications, readmissions, or emergency department (ED) visits.^4,5,6,7,8,9,10,11,12,13,14,15,16,17,18,19^ Furthermore, a recent large database study found that contemporary outpatient TJA is associated with fewer adverse events when matched to inpatient procedures.^20^ Compared with inpatient TJA, patients who undergo outpatient TJA may also have higher satisfaction and improved patient-reported outcomes.^10,21^

Safety net hospitals, such as most government-funded county facilities in the United States, treat a large proportion of patients without insurance or with Medicaid.^22^ Therefore, these facilities tend to serve vulnerable populations that have the least access to health care, yet often have the highest severity of illnesses, surgical costs, hospital length of stay (LOS), surgical mortality, complications, and readmission rates.^23^ These populations also tend to have higher rates of hip and knee osteoarthritis^24^ and pose a unique challenge in TJA due to lower general health maintenance and medical optimization, higher substance abuse rates, lower socioeconomic status, and oftentimes less reliable home and social support systems.^24,25,26,27^ Further contributing to this challenge, or perhaps as a result of it, this patient population has been shown to have decreased satisfaction, worse outcomes, and increased complications after TJA.^26,27,28,29,30^

With growing evidence that outpatient TJA can be performed safely and effectively while drastically lowering economic costs in the general population,^4,17-19,25^ our goal was to adopt this process at a safety net county hospital. Transitioning to outpatient TJA in this setting could provide equal opportunities in care for vulnerable populations while also reducing the economic burden of TJA in a government-funded hospital system.

In May 2020, a single surgeon at our safety net county hospital instituted an outpatient TJA protocol for all primary arthroplasty patients. The purpose of this study was to report on the feasibility of such a program with regard to rates of same-day discharge (SDD), hospital LOS, and adverse events. Our hypothesis is that outpatient arthroplasty can be safely and effectively performed in a safety net hospital system (SNHS) without increasing short-term complications, readmissions, revision surgeries, or ED visit rates.

## Background

In 2019, our institution hired a new adult reconstruction fellowship-trained orthopaedic surgeon. He instituted changes to the arthroplasty service line that focused on rapid recovery and early discharge for all TJA patients. This rapid recovery protocol (RRP) involved optimizing medical conditions and social circumstances preoperatively, increasing patient and family education, instituting perioperative changes including the increased use of spinal anesthesia and a multimodal pain regimen that minimizes narcotic use, routine use of tranexamic acid, avoiding routine use of closed suction drains or indwelling urinary catheters, and encouraging early mobilization with physical therapy (PT) and occupational therapy (OT).

When instituting these changes for the RRP, outpatient arthroplasty was always the goal. However, neither next-day discharge (NDD) nor outpatient TJA had previously been performed in this healthcare system, and there were, therefore, several challenges during this transition. The RRP was first instituted in October 2019 with the goal of NDD. After several months of success with the RRP and NDD, in May 2020, an interdisciplinary agreement was made among the surgeons, anesthesia providers, perioperative teams, PT, OT, nursing, case management, social work, and hospital administrators to allow for possible same-day discharge (PSDD) outpatient arthroplasty for patients who met all required postoperative goals of discharge on the day of surgery.

## Methods

Institutional review board approval was obtained for this retrospective review of all primary TJA performed by a single surgeon (RR) from October 1, 2019, to October 31, 2020. All patients undergoing primary unilateral total knee arthroplasty (TKA) and total hip arthroplasty (THA) based on current procedural terminology codes 27447 (primary TKA), 27130 (primary THA), and 27132 (conversion THA) were included. Throughout the study period, patient selection criteria remained consistent with our institution's expected practice guidelines for elective primary TJA and were not changed when the transition to outpatient TJA was made. Exclusion criteria for patient selection included body mass index greater than 40 kg/mg^2^, hemoglobin A1C greater than 8%, uncontrolled axis 1 or 2 psychiatric disease, severe aortic stenosis or pulmonary hypertension, open leg ulcerations, intravenous drug use within 1 year, active nicotine use, Child class B or C liver disease, narcotic use greater than 40 morphine milliequivalents per day, and current homelessness. There were no exclusions for degree of deformity or complexity of the reconstruction. In addition to patient selection criteria, exclusion criteria for the present study also included revision TJA and same-day bilateral simultaneous TJA.

The patients were divided into two cohorts for the purposes of this study. The first group included all patients treated with TJA from October 1, 2019, to April 30, 2020. All patients in this group were under the RRP with the goal of NDD. The second group included all patients treated from May 1, 2020, to October 31, 2020, who were also in the RRP but now had the option of PSDD. Before discharge for both groups, certain criteria had to be met: receiving clearance from OT and PT for safe discharge home, tolerating an oral diet, urinating independently, having their pain controlled on an oral medication regimen, and demonstrating stable vital signs.

Demographic and perioperative data were collected through detailed retrospective chart review. Patients who underwent staged bilateral TJA with separate hospitalizations had each procedure examined as an independent event. The American Society of Anesthesiologists Physical Status classification system is a tool that uses preoperative medical comorbidities to assess perioperative risks^31^ and was identified in the preoperative anesthesia evaluation note. The Charlson Comorbidity Index is a tool used to predict 10-year survival in patients with multiple medical comorbidities and was calculated based on *International Classification of Diseases* diagnosis codes in the standard proposed fashion.^32,33^ The type of anesthesia used was examined and recorded as either general endotracheal or spinal anesthesia. Although the goal was to use spinal anesthesia in all cases, general anesthesia was used at the discretion of the anesthesiologist if spinal anesthesia was unable to be successfully administered. There were no narcotics in any spinal injections. Patients received a single shot of either a hyperbaric bupivacaine injection for TKA or isobaric bupivacaine injection for THA (both without narcotics). The specific volume of bupivacaine for each case was between 1.4 and 2.0 mL.

The primary outcome measure evaluated was the rate of successful SDD in the PSDD cohort. Secondary outcome measures included hospital LOS, discharge destination, 30-day ED visits, 30- and 90-day complications and readmissions, and 90-day revision surgeries.

Data were collected using Microsoft Excel and analyzed using IBM SPSS Statistics (version 10.15 for macOS) using a two-sided level of significance of 0.05. All continuous variables were analyzed via unpaired *t* tests, and all categorical data were analyzed via Chi-square or Fisher exact tests.

## Results

Initial query yielded 122 consecutive primary TJAs performed during the study period. Four procedures in two patients were same-day bilateral surgeries and were therefore excluded, resulting in a total of 118 cases for analysis. Of these, 57 were performed before the possibility of SDD and were therefore considered as the NDD cohort, and the subsequent 61 were performed with the possibility of SDD and were therefore considered as the PSDD cohort. All patients had a minimum 90-day follow-up, with no patients being lost to follow-up in this series.

The cohorts were similar with no statistical differences in type of procedure, side of surgery, age, sex, race, primary language spoken, body mass index, inflammatory arthritis versus osteoarthritis, presence of diabetes mellitus, smoking status, American Society of Anesthesiologists Physical Status score, Charlson Comorbidity Index, indwelling urinary catheter use, or estimated blood loss (Table 1). In the PSDD cohort, mean HbA1c in patients with diabetes was statistically significantly greater (6.7 versus 6.5, *P* = 0.0075), and spinal anesthesia was performed at a significantly higher rate (100% versus 87.7%, *P* = 0.005).

**Table 1:** Comparison of Cohort Demographics and Perioperative Variables

Characteristic	Next-Day Discharge (NDD) Cohort		PSDD Cohort		*P*
Total TJAs performed	57		61		
Operation type					0.36
TKA	48	84.2%	47	77.0%	
THA	9	15.8%	14	23.0%	
Side of surgery					0.28
Right	32	56.1%	28	45.9%	
Left	25	43.9%	33	54.1%	
Age (yr)	61.1 ± 11.6 (29-83)		60.6 ± 11.4 (24 - 77)		0.81
Sex					0.69
Male	17	29.8%	16	26.2%	
Female	40	70.2%	45	73.8%	
Race					0.89
Hispanic	40	70.2%	47	77.0%	
Black	10	17.5%	9	14.8%	
White	4	7.0%	2	3.3%	
Asian	2	3.5%	2	3.3%	
Other	1	1.8%	1	1.6%	
Primary language					0.83
English	13	22.8%	15	24.6%	
Not English	44	77.2%	46	75.4%	
BMI (Kg/m^2^)	31.0 ± 4.4 (21.2-40.1)		30.9 ± 4.7 (19.6-43.5)		0.96
Type of arthritis					0.29
Inflammatory	10	17.5%	6	9.8%	
Noninflammatory	47	82.5%	55	90.2%	
DM	10	17.5%	18	29.5%	0.14
Preoperative Hb A1C	6.5 ± 0.5 (6.0-7.4)		6.7 ± 0.5 (5.8-7.7)		0.0075^a^
Smoking status					
Never	48	84.2%	47	77.0%	0.36
Former	9	15.8%	14	23.0%	0.36
Current	0	0%	0	0%	1.0
ASA-PS score	2.46 ± 0.54 (1-3)		2.48 ± 0.54 (1-3)		0.85
CCI	2.5 ± 1.4 (0-5)		2.6 ± 1.6 (0-9)		0.76
Anesthesia type					0.005^a^
Spinal	50	87.7%	61	100%	
General endotracheal	7	12.3%	0	0%	
Indwelling urinary catheter	3	5.3%	0	0%	0.11
Estimated blood loss (mL)	96.8 ± 75.6 (20-400)		106.1 ± 94.3 (15-500)		0.56

ASA-PS = American Society of Anesthesiologists Physical Status, BMI = body mass index, CCI = Charlson Comorbidity Index, DM = diabetes mellitus, Hb A1C = hemoglobin A1C, NDD = next-day discharge, PSDD = possibility of same-day discharge, THA = total hip arthroplasty, TJA = total joint arthroplasty, TKA = total knee arthroplasty ^a^*P* < 0.05.

X ± Y (A − B); mean ± SD (range).

In the PSDD group, 86.9% (53/61) of patients were successfully discharged on postoperative day (POD) 0, with the remaining 13.1% (8/61) discharged on POD 1 (Table 2). Day of discharge was significantly different when compared with the NDD cohort in which 100% (57/57) of the patients were discharged on POD 1 (*P* < 0.0001). The mean hospital LOS was reduced from 31.2 hours in the NDD cohort to 13.5 hours in the PSDD cohort (*P* < 0.0001), a mean difference of 17.7 hours. All of the patients in both cohorts were discharged to home.

**Table 2:** Comparison of Hospital Length of Stay and Postoperative Day of Discharge

Variables	NDD Cohort		PSDD Cohort		*P*
Total TJAs performed	57		61		
Average hospital LOS					<0.0001^a^
Days	1.30 ± 0.11 (0.93-1.67)		0.56 ± 0.27 (0.19-1.33)		
Hours	31.2 ± 2.7 (22.2-40.0)		13.5 ± 6.5 (4.5-31.9)		
Day of discharge					<0.0001^a^
POD 0	0	0%	53	86.9%	
POD 1	57	100%	8	13.1%	
POD ≥ 2	0	0%	0	0%	

LOS = length of stay, NDD = next-day discharge, POD = postoperative day, PSDD = possibility of same-day discharge, TJA = total joint arthroplasty

a *P* < 0.05.

X ± Y (A − B); mean ± SD (range).

When comparing NDD versus PSDD patients (Table 3), no significant difference was found in 30-day complications (3.5% versus 4.9%, *P* = 1.0), 90-day complications (15.8% versus 13.1%, *P* = 0.79), 30-day readmissions (0% versus 1.6%, *P* = 1.0), 90-day readmissions (0% versus 3.3%, *P* = 0.50), 90-day revision surgeries (0% versus 3.3%, *P* = 0.50), or 30-day ED visits (5.3% versus 3.3%, *P* = 0.67). There was also no significant difference in number of acute medical complications (1.8% versus 3.3%, *P* = 1.0), acute surgical complications (14% versus 9.8%, *P* = 1.0), superficial wound complications (14% versus 8.2%, *P* = 0.38), or deep wound complications (0% versus 1.6%, *P* = 1.0).

**Table 3:** Comparison of Short-term Adverse Events

Variables	NDD Cohort		PSDD Cohort		*P*
Total TJAs performed	57		61		
Length of recorded follow-up (d)	273.5 ± 85.3 (132-445)		129.9 ± 31.8 (90-232)		<0.0001^a^
30-day complications	2	3.5%	3	4.9%	1.0
90-day complications	9	15.8%	8	13.1%	0.79
Acute medical complications	1	1.8%	2	3.3%	1.0
Acute surgical complications	8	14%	6	9.8%	1.0
Superficial wound complications	8	14%	5	8.2%	0.38
Deep wound complications	0	0%	1	1.6%	1.0
30-day readmissions	0	0%	1	1.6%	1.0
90-day readmissions	0	0%	2	3.3%	0.50
30-day ED visits	3	5.3%	2	3.3%	0.67
90-day revision surgeries	0	0%	2	3.3%	0.50

ED = emergency department, NDD = next-day discharge, PSDD = possibility of same-day discharge, TJA = total joint arthroplasty ^a^*P* < 0.05.

X ± Y (A − B); mean ± SD (range).

**Figure 1:**
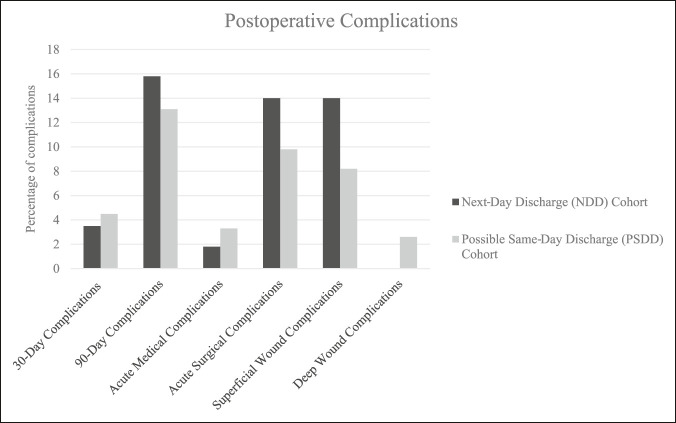
Histogram illustrating the percentage of patients in each cohort with each type of complication. No deep wound complications were found in the next-day discharge (NDD) cohort.

In the NDD cohort, the nine complications included one acute medical complication (1.8%) involving a patient who experienced postoperative hypotension and bradycardia determined to be due to a vasovagal response and eight acute surgical complications (14%), all of which were superficial wound complications that went on to heal with local wound care (Figure 1). There were no readmissions or revision surgeries. The three ED visits (5.3%) involved a patient presenting on POD 5 due to postoperative pain and swelling determined by the lead surgeon to be consistent with standard postoperative pain, a patient presenting on POD 3 with transient confusion that yielded a negative workup in the ED that resolved shortly thereafter, and a patient presenting on POD 26 with incidental tooth pain that was subsequently referred to a dental clinic.

In the PSDD cohort, the eight complications included 2 acute medical complications (3.3%): one patient diagnosed with a pulmonary embolism on POD 11, which was thereafter managed on XARALTO (rivaroxaban) without further complication, and one TKA patient who presented with sepsis on POD 22 that was found to have a retroperitoneal abscess, which was successfully treated with interventional radiology-guided drainage and antibiotics. The six remaining complications were acute surgical complications (9.8%) including five superficial wound complications (8.2%) and one deep wound complication (1.6%) (Figure 1). There were two revision surgeries (3.3%): one TKA that required irrigation and débridement with polyethylene insert exchange for acute periprosthetic joint infection (PJI) on POD 28 and one TKA that required irrigation and débridement of skin, subcutaneous tissue, fat, and fascia for a wound dehiscence superficial to the intact arthrotomy that was not consistent with PJI on POD 41. There were two readmissions (3.3%): one for the abovementioned patient presenting with sepsis on POD 22 and one for the patient with PJI admitted after the revision surgery on POD 28. The two ED visits (3.3%) involved the abovementioned patients presenting with sepsis on POD 22 and pulmonary embolism on POD 11.

The NDD cohort had a significantly longer average follow-up duration as a result of these operations occurring chronologically sooner than those in the PSDD cohort (273.5 versus 129.9 days, *P* < 0.0001) (Table 3).

## Discussion

Our early results suggest that outpatient TJA can be safely and effectively performed in an SNHS. We found that by transitioning our goal day of discharge from POD 1 to POD 0, the average LOS was decreased by 17.2 hours and that 86.9% of patients were able to be discharged on the same day of surgery without increasing short-term rates of complications, readmissions, revision surgeries, or ED visits.

Outpatient TJA has previously been shown to provide an opportunity to substantially decrease hospital and procedural-related costs^4,18,19^ without increasing rates of complications, readmissions, or ED visits.^5,6,7,8,9,10,11,12,13,14,15,16,17^ More recently, a large database study has demonstrated that inpatient arthroplasty is an independent risk factor for higher rates of adverse events when propensity score matched to outpatient arthroplasty for TKA, THA, and unicompartmental knee arthroplasty.^20^ However, these studies did not specifically examine marginalized patients such as those evaluated in this study that have been shown to have decreased satisfaction, worse outcomes, and increased complications after TJA.^26,27,28,29,30^ Thus, before transitioning to outpatient TJA at our county hospital, we were unsure whether the challenges unique to this patient population would threaten patient outcomes. If outpatient TJA in an SNHS is to both benefit the patients and reduce the economic burden to society, it must be done so without increasing adverse events that lead to inferior outcomes and secondary increases in costs for ED visits, readmissions, or revision surgeries.

Quantifying the exact cost savings of outpatient versus NDD arthroplasty can be challenging given variations in healthcare systems, locations, patient populations, and surgeon preferences. Although the cost savings were not quantified in the present study, previous studies have shown that outpatient TJA can result in significant overall cost reductions when comparted with inpatient TJA. In a single-surgeon case-control study, Aynardi et al^4^ noted a mean cost savings of $6798 when comparing 119 outpatient with 78 inpatient THAs. Huang et al^18^ performed a similar single-surgeon case-control study evaluating 20 outpatient versus 20 inpatient TKAs and noted median cost savings of 30%. In a large Medicare database study, Lovald et al^19^ found 2-year osteoarthritis attributable costs to be approximately $6500 higher for 1 to 2 day stay TKAs (7755 patients) when compared with outpatient TKAs (454 patients). Limited data exist specifically examining cost savings of SDD versus NDD TJA.

A major concern for surgeons transitioning to outpatient TJA is that patients will sustain early complications at home or return to the ED for medical complications that could have otherwise been managed in the hospital had they been admitted postoperatively. In our study, no patients in the PSDD cohort and therefore no patients discharged on the same day of surgery experienced a complication or unanticipated care episode within the first week postoperatively. The absence of 1-week postoperative complications and unanticipated care episodes in these patients strengthens the argument that outpatient TJA can be performed safely in this patient population without increasing secondary costs due to unanticipated care episodes.

Intuitively, as patients were allowed for PSDD, a significant decrease was found in LOS and POD of discharge. However, there were 8 cases within the PSDD cohort in which patients failed to be discharged POD 0, all of which were due to the inability to clear PT. Seven of these occurred in THA patients, three of which involved bulk femoral head autografting for acetabular reconstruction in severe dysplasia. At our institution, these structural autograft THA patients are made protected weight bearing in the initial postoperative period, which may have hindered their ability to mobilize with PT on POD 0. Interestingly, in the PSDD cohort, both patients who experienced 30-day ED visits, one of the 30-day complications, and one of the 30-day readmissions were patients who initially failed to be discharged on POD 0 and instead were discharged on POD 1.

Studies outside of the United States have demonstrated that outpatient TKA^34^ and THA^35^ performed in government-funded healthcare systems can be safe and effective with comparable early adverse event rates to inpatient procedures. To our knowledge, however, there has only been one previous publication that reported on outpatient TJA in an SNHS. In their study, Schultz et al^25^ evaluated patients who underwent primary TJA before and after implementation of an accelerated recovery program and found that the patients treated after implementation of the program had decreased hospital LOS, increased discharges to home, decreased procedure and hospitalization costs, and fewer complications, with an SDD rate of 6.48% (7/108 patients). Our SDD rate in the PSDD cohort of 86.9% (53/61 patients) builds on their findings and shows that it is not only possible to perform outpatient TJA in an SNHS, but it is possible to do so for most patients in which it is attempted. Our SDD rate of 86.9% approaches the rate of 94.7% (955 of 1009 patients) presented in a recent systematic review that evaluated patients in which outpatient TJA was planned.^6^

A significant factor in the success of our outpatient TJA program is the dedicated patient and family education that is provided throughout the preoperative, perioperative, and postoperative period. Starting at their initial clinic visit, patients and family members are prepared to expect early discharge and educated on how to optimize their home environment and recovery once out of the hospital. For all outpatient cases, patients are called by the lead surgeon (RR) on POD 1 to check in on them and answer any additional questions they might have, potentially decreasing the number of unnecessary ED visits.

For English-speaking healthcare providers, delivering equal and adequate patient education within our institution can be a challenge given the diversity of cultural backgrounds and languages in the region. With only 23.7% (28/118) of our patients speaking English as their first language, reliance on interpretive services is key for communication. Our study shows that even in this patient population, TJA, a procedure that relies heavily on patient and family education and participation, can be performed safely as an outpatient procedure. Another key to success has been the communication and resources of multidisciplinary collaboration alongside PT, OT, anesthesiology, and internal medicine specialists to take the leap from NDD to SDD TJA.

This study has limitations including those inherent to retrospective review of a single surgeon's experience. Our results may not be generalizable for facilities in which the resources required for such an endeavor are not adequate. Functional or patient-reported outcome measures and the cost savings of SDD compared with NDD TJA were not evaluated in this study and may be investigated in the future. Last, as a retrospective study, there are limitations in the quality of the data that can be obtained, and there is the possibility that certain events were not documented in our records if managed at outside institutions. However, because of insurance status, most patients treated at our institution are only able to receive care within our single health system and single electronic medical record system. Fortunately, all patients included in the current study had greater than 90-day follow-up, with no patients being lost to follow-up, which strengthens the quality of our findings. Despite the size and limitations of our study, we feel that the results presented on our successful transition to outpatient TJA in an SNHS is significant enough to be impactful to other safety net or county facilities considering the transition to outpatient TJA. Future studies to further investigate short- and long-term adverse event rates, patient-reported outcome measures, and cost savings are planned.

## Conclusion

The purpose of this study was to report on the feasibility of performing high-percentage outpatient THA and TKA in an SNHS. We have demonstrated that the transition to outpatient arthroplasty can be safely and effectively performed in up to 86.9% of patients in an SNHS without significantly increasing short-term complications, readmissions, revision surgeries, or ED visit rates.
